# Whole-genome sequencing of extrachromosomal circular DNA of cerebrospinal fluid of medulloblastoma

**DOI:** 10.3389/fonc.2022.934159

**Published:** 2022-11-08

**Authors:** Yi Zhu, Zhihui Liu, Yuduo Guo, Shenglun Li, Yanming Qu, Lin Dai, Yujia Chen, Weihai Ning, Hongwei Zhang, Lixin Ma

**Affiliations:** ^1^ Department of Neurosurgery, Binzhou Medical University Hospital, Binzhou, China; ^2^ Department of Obstetrics and Gynecology, Beijing Chaoyang Hospital, Capital Medical University, Beijing, China; ^3^ Chinese Academy of Sciences (CAS) Key Laboratory of Infection and Immunity, Institute of biophysics, Chinese Academy of Sciences, Beijing, China; ^4^ Department of Neurosurgery, Sanbo Brain Hospital, Capital Medical University, Beijing, China; ^5^ Department of Neurosurgery, Beijing Chaoyang Hospital, Capital Medical University, Beijing, China

**Keywords:** extrachromosomal circular DNAs (eccDNAs), medulloblastoma (MB), liquid biopsy, differentially expressed genes, GEO

## Abstract

**Background:**

Medulloblastoma (MB) is a malignant tumor associated with a poor prognosis in part due to a lack of effective detection methods. Extrachromosomal circular DNA (eccDNA) has been associated with multiple tumors. Nonetheless, little is currently known on eccDNA in MB.

**Methods:**

Genomic features of eccDNAs were identified in MB tissues and matched cerebrospinal fluid (CSF) and compared with corresponding normal samples using Circle map. The nucleotides on both sides of the eccDNAs’ breakpoint were analyzed to understand the mechanisms of eccDNA formation. Bioinformatics analysis combined with the Gene Expression Omnibus (GEO) database identified features of eccDNA-related genes in MB. Lasso Cox regression model, univariate and multivariate Cox regression analysis, time-dependent ROC, and Kaplan–Meier curve were used to assess the potential diagnostic and prognostic value of the hub genes.

**Results:**

EccDNA was profiled in matched tumor and CSF samples from MB patients, and control, eccDNA-related genes enriched in MB were identified. The distribution of eccDNAs in the genome was closely related to gene density and the mechanism of eccDNA formation was evaluated. EccDNAs in CSF exhibited similar distribution with matched MB tissues but were differentially expressed between tumor and normal. Ten hub genes prominent in both the eccDNA dataset and the GEO database were selected to classify MB patients to either high- or low-risk groups, and a prognostic nomogram was thus established.

**Conclusions:**

This study provides preliminary evidence of the characteristics and formation mechanism of eccDNAs in MB and CSF. Importantly, eccDNA-associated hub genes in CSF could be used as diagnostic and prognostic biomarkers for MB.

## Introduction

Medulloblastoma (MB) is one of the most common malignant tumors of the central nervous system (CNS) in children, with an annual incidence of about five cases per 1 million people (1), and an overall 5-year survival rate of 70%–85% in the standard-risk group (2, 3) and less than 30% in the high-risk group (4, 5). The diagnosis of MB is mainly based on clinical symptoms, imaging findings, cerebrospinal fluid (CSF) examination, and histopathological examination. With the addition of molecular pathology, the 2016 edition of the World Health Organization (WHO) CNS tumor classification classifies MB into four subgroups (6): wingless pathway (WNT), sonic hedgehog (SHH), Group 3, and Group 4, making the diagnosis and treatment of MB more individualized. However, this raises the demand for accurate detection tools to select the optimal treatment regimen and assess treatment response and monitor relapse. Currently, clinical monitoring of MB is commonly done by magnetic resonance imaging (MRI) and CSF cytology, but the sensitivity of these two methods can be limited by the extent of tumor growth and affect the assessment of the disease (7, 8). Accordingly, there is an urgent clinical need for a more sensitive way to reliably monitor tumor status that may not have changed on imaging.

Extrachromosomal circular DNA (eccDNA) is a type of circular DNA located outside of chromosomes, independent of the traditional genome structure previously thought. Wu et al. published electron microscopy photographs of eccDNA and supported the widespread presence of eccDNA in human tumor cells and normal tissues in 2019 (9). Several studies published subsequently revealed the unique topological structure and genetic properties of eccDNA, which can rapidly remodel the genome through diversity (including structural, functional, and quantitative diversity) and thus are directly and effectively involved in cancer development (9–13). An increasing body of evidence suggests that eccDNAs can be derived from multiple genes and contain one or more gene fragments, intact genes, or regulatory regions; in tumors, eccDNA contains oncogenes or genes associated with drug resistance in cancer therapy (10, 14), tumor heterogeneity, and adaptability. In addition, eccDNA leads to an increase in the copy number of oncogenes (15), resulting in high levels of oncogene products; indeed, oncogene amplification on eccDNA is significantly more efficient than on chromosomes (10, 16). Current evidence suggests that eccDNA abundance is significantly associated with cancer progression and poor prognosis in various tumors (16). Taken together, the above findings indicate the great potential of eccDNA in cancer therapy.

Ana C. de Carvalho et al. found that the regulation of epidermal growth factor receptor (EGFR) VIII expression by eccDNA in glioblastoma (GBM) is significantly associated with resistance to EGFR inhibitors (11). Another report found that eccDNA is involved in and promotes most genomic rearrangements in neuroblastoma that induce mutant phenotypes, leading to tumor development and affecting patient survival (10). It is highly conceivable that eccDNA in MB may have some hitherto unexplored but important functions and molecular mechanisms. That eccDNA has potential in clinical diagnosis is demonstrated by the fact that fetal- and maternal-derived eccDNAs exist simultaneously in the plasma of pregnant women, with significant differences in fragment size and chromosome distribution (17), Similarly, eccDNA also holds promise in cancer diagnostics because eccDNA could be released from tumors (18). Importantly, eccDNAs are more stable than linear DNAs in blood circulation, suggesting that eccDNAs have the potential for clinical application as a novel cancer biomarker in liquid biopsies (19).

Considering the importance of eccDNA in cancer, here we investigated the mechanism of eccDNA formation in MB and the diagnostic potential of profiling eccDNA in CSF samples. We reasoned that, for diagnostic purposes, CSF samples are superior to both MB tissue and plasma samples, are relatively easy to obtain, and are in direct contact with MB tissue. For this purpose, eccDNA was profiled in matched tumor and CSF samples from *n* = 3 MB samples and *n* = 1 control; one separate MB tissue was also included in the MB group. To classify MB patients into high- or low-risk groups, 10 hub genes prominent in both our eccDNA data and gene expression profiles in high- and low-risk MB patients as well as controls from databases were selected. Finally, the selected genes were used to establish a prognostic nomogram and evaluate the diagnostic potential of eccDNA in MB.

## Materials and methods

### Sample collection and DNA preparation

MB tissue samples and matched CSF samples were obtained from four patients who underwent surgery at the Department of Neurosurgery, Sanbo Brain Hospital, Capital Medical University (Beijing, China) between January 2020 and November 2021, with a pathological diagnosis of MB. After harvesting, the tumor tissues were rapidly frozen in liquid nitrogen and stored in a −80°C refrigerator. CSF was processed using a standardized protocol and immediately stored in a −80°C refrigerator. All patients provided informed consent, and the protocols used in this study were approved by the local institutional review board.

### Sequencing and analysis of eccDNA

The eccDNA extraction, enrichment, and amplification procedures were conducted as previously described in the literature with slight modifications (19, 20). DNA quantification and detection of DNA integrity were performed by a Qubit 3.0 fluorometer (**Table S1**) and agarose gel electrophoresis (**Figure S1A**), respectively. Agilent 2100 Bioanalyzer (Agilent Technologies, Inc., USA) was used to determine the library quality (**Figure S1B**) (**Table S2**). Circle-Seq and Circulome-seq eccDNA sequencing Service was provided by CloudSeq Biotech Inc. (Shanghai, China).

(1) Tissue DNA Library Preparation and Sequencing (19): Tissue samples (6 mg) were placed in a 600-µl L1 buffer (Plasmid Mini AX; A&A Biotechnology: #010-50), and 15 µl of Proteinase K (ThermoFisher: #4333793) was added for incubation overnight at 50°C. The lysed samples were alkaline treated and purified through an ion exchange membrane column, according to the instructions (Plasmid Mini AX; A&A Biotechnology). Column-purified DNAs were digested for 16 h by FastDigest MssI (Thermo Scientific: #FD1344) at 37°C to remove mitochondrial circular DNA, as recommended by the manufacturer’s protocol. DNAs were then incubated at 37°C with exonuclease (Plasmid-Safe ATP-dependent DNase, Epicentre: #E3101K); additional ATP (2 µl) and DNase (2.5 µl) were added every 24 h continuously for 1 week to remove the remaining linear DNA, as recommended by the manufacturer’s protocol (Plasmid-Safe ATP-dependent DNase, Epicentre). The processed DNAs were used as a template for eccDNA amplification *via* phi29 polymerase reactions (REPLI-g Midi Kit, Qiagen: #150043) at 30°C for 2 days (46–48 h), as recommended by the manufacturer’s protocol. Amplified DNAs were sheared with sonication (Bioruptor), and the sequencing libraries were prepared using the NEBNext^®^ Ultra II DNA Library Prep Kit for Illumina (New England Biolabs; #E7645) following the manufacturer’s manual. Sequencing was performed on Illumina NovaSeq 6000 with 150-bp paired-end mode according to the manufacturer’s instructions.

(2) DNA of CSF library preparation and sequencing (20): The QIAamp Circulating Nucleic Acid Kit (Qiagen: #55114) was used to extract DNA in CSF of four samples (1 ml/sample), as recommended by the manufacturer’s protocol. To remove linear DNA, the DNA was digested for 5 min with exonuclease V (New England Biolabs: #M0345S) at 37°C, as recommended by the manufacturer’s protocol. The circular structure of eccDNA was opened by transposable enzymes, and the ends of the DNA fragments were attached to the joints. Next, the Klenow enzyme (New England Biolabs: #M0210L) was used to fill these gaps and ends, as recommended by the manufacturer’s protocol. Then, these products were amplified and purified by PCR. The sequencing libraries were prepared with the NEBNext^®^ Ultra™ DNA Library Prep Kit (New England Biolabs: #E7645S) following the manufacturer’s manual. Agilent 2100 Bioanalyzer (Agilent Technologies, Inc., USA) was used to determine the library quality. DNA libraries were sequenced on Illumina NovaSeq 6000 with 150-bp paired-end mode according to the manufacturer’s instructions.

Quantitative PCR was performed with SYBR Premix Ex Taq (Takara: #RR420A) under conditions of 40 cycles of PCR, as recommended by the manufacturer’s protocol. COX5B was amplified with the forward primer GGGCACCATTTTCCTTGATCAT and reverse primer AGTCGCCTGCTCTTCATCAG. Paired-end reads were obtained from the Illumina NovaSeq 6000 sequencer and were quality controlled by Q30 (**Table S3**). After 3’ adaptor-trimming and low-quality read removal by cutadapt software (v1.9.1) (21), the high-quality clean reads were aligned to the reference genome with Burrows–Wheeler Alignment (BWA) software (v0.7.12) (22). Then, Circle-Map (v1.1.4) (23) was used to detect eccDNA within all samples, and Samtools (v0.2) (24) was used to obtain raw soft-clipped read counts of the breakpoint. Then, edgeR (25) (v0.6.9) was used to perform normalization and differentially expressed eccDNA filter by *p*-value and fold change. Bedtools (v2.27.1) (26) was used to annotate the eccDNAs. IGV (27) software (v2.4.10) was used for eccDNA visualization.

### Gene enrichment analysis

To better understand the functions of the known or predicted genes, Gene Ontology (GO) analysis was conducted in terms of biological process (BP), cellular component (CC), and molecular function (MF) by the “clusterProfiler” R package (4.0.5). The “clusterProfiler” was used to understand the relationship between genes and pathways provided by the Kyoto Encyclopedia of Genes and Genomes (KEGG) pathway database (28).

### Acquisition of gene expression and clinical data

The patient’s data for MB tissues and normal brain tissues gene expression and platform profiles of GSE85217 (29) and GSE124814 (30) were downloaded from the National Center for Biotechnology Information Gene Expression Omnibus (NCBI-GEO) (http://www.ncbi.nlm.nih.gov/geo). According to the annotation information on the platform, the probes were converted into corresponding gene symbols. A total of 337 patients with clinical survival and follow-up information from dataset GSE85217 were included in the survival analysis as the training cohort.

### Construction of the gene signature model and validation

The univariate analysis was performed by the “survival” and “survminer” R packages (https://github.com/therneau/survival) (https://github.com/kassambara/survminer) to identify OS-related hub genes. Lasso-penalized Cox regression analysis (31) was performed to construct the prognostic gene signature. The prognostic gene signature was presented as a risk score obtained by the “survival” R package. Taking the median risk score as the cutoff value, 337 patients were divided into high- and low-risk groups. Kaplan–Meier (KM) survival curves and time-dependent receiver operational feature (ROC) curve analyses were generated to assess the predictive capacity of the model (32). Univariate and multivariate Cox regression analyses were performed to evaluate the survival status. The hazard ratio (HR) and 95% confidence interval (CI) were calculated to identify genes related to overall survival (OS). The area under the curve (AUC) of the ROC was used to compare the diagnostic and prognostic abilities of different indexes. All independent prognostic parameters and corresponding clinical data were included in a prognostic nomogram constructed by a stepwise Cox regression model to predict the 3-, 5-, and 10-year OS of MB patients in the training set.

### Statistical analysis

All statistical analyses were performed by R software version 4.1.2 and visualized by GraphPad Prism 8.0 (GraphPad Software, La Jolla, CA, USA) and the “ggplot” R package (https://ggplot2.tidyverse.org/). Average and standard deviations were calculated for all data; as the underlying data distribution was unknown, Wilcoxon rank-sum test and Friedman test were applied to compare data from two or more groups using GraphPad Prism 8.0. In the enrichment analysis, the Benjamini and Hochberg (1995) test (33) has been applied to evaluate GO ID; a false discovery rate (FDR) < 0.25 and adj.p <0.05 were considered statistically significant. Lasso-penalized Cox regression analysis was performed to construct the prognostic gene signature. The KM method was used to compare OS between the two groups, and the logarithmic rank test was used to assess the difference in survival curves. Univariate and multivariate Cox regression analyses were performed to evaluate the survival status, and the hazard ratio (HR) and 95% confidence interval (CI) estimated the hazard risk of the individual indicators. *p*-value < 0.05 was statistically significant unless otherwise specified. * represents *p* < 0.05, ** represents *p* < 0.01, and *** represents *p* < 0.001.

## Results

### Identification and verification of eccDNAs from tissue and CSF

**Figure 1:**
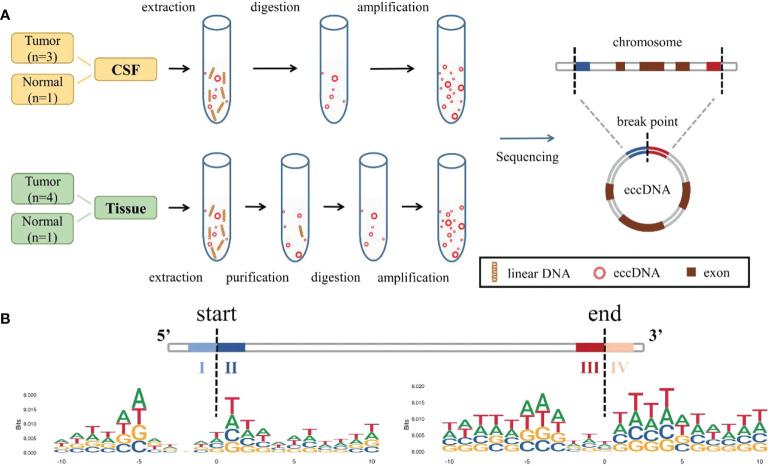
Identification and mapping process of eccDNAs in samples. **(A)** Two different methods were used to extract, enrich, and amplify eccDNAs from MB tissue samples and CSF samples, respectively, then compared to the reference genome. **(B)** Trinucleotide motif sequences flanking the start and end positions of eccDNA in normal CSF were labeled as I, II, III, and IV. eccDNAs, extrachromosomal circular DNAs; MB, medulloblastoma; CSF, cerebrospinal fluid.

We adopted two different processes based on the Circle-Seq (19, 34, 35) method for better extraction and enrichment of eccDNAs in tissue and CSF, respectively. The samples were divided into two groups, one with tissue and matched CSF from MB (*n* = 3) and the other with normal (*n* = 1); one separate MB tissue sample was also included in the MB group. For tissue samples, column separation of eccDNA was used and incubated with exonuclease for better removal of linear genomic DNA. Then, the products were rolling-circle amplified before being sheared by sonication, and the fragmented DNAs were later used for library preparation for next-generation sequencing. For CSF samples, linear DNAs were removed directly using exonuclease V (Exo V), the circular structure of eccDNAs was opened by transposable enzyme, and the Klenow enzyme was used for gap/end repair. Finally, a polymerase chain reaction (PCR) was performed to amplify and purify the products for sequencing. Referring to Circle-Seq (19), COX5B, a gene absent from eccDNA, was measured using quantitative PCR (qPCR) to verify that linear DNA was removed after exonuclease treatment (**Table S3**). The overall process is shown in **Figure 1A**. Original reads were quality controlled by Q30 (**Table S4**), low-quality reads were removed, and the high-quality clean reads were aligned to the reference genome with BWA (22) (**Table S5**). Then, Circle-Map (23) software was used to detect eccDNA within all samples and obtain raw soft-clipped read counts of the breakpoint. Finally, more than 30,000 different eccDNAs (average: 6,718; median: 5,472) were identified from nine samples.

We explored the possible mechanism of eccDNA formation by analyzing DNA sequences from 10 bp upstream to 10 bp downstream of the start and end positions of each eccDNA; the eccDNA sequences were acquired from our data, while sequences besides the eccDNA sequences were inferred from the reference genome. Trinucleotide motif sequences flanking the start and end positions of eccDNA in each group were labeled as I, II, III, and IV, respectively (**Figure 1B**, **Figures S2A–C**).

### Detection and analysis of eccDNAs in different samples

**Figure 2:**
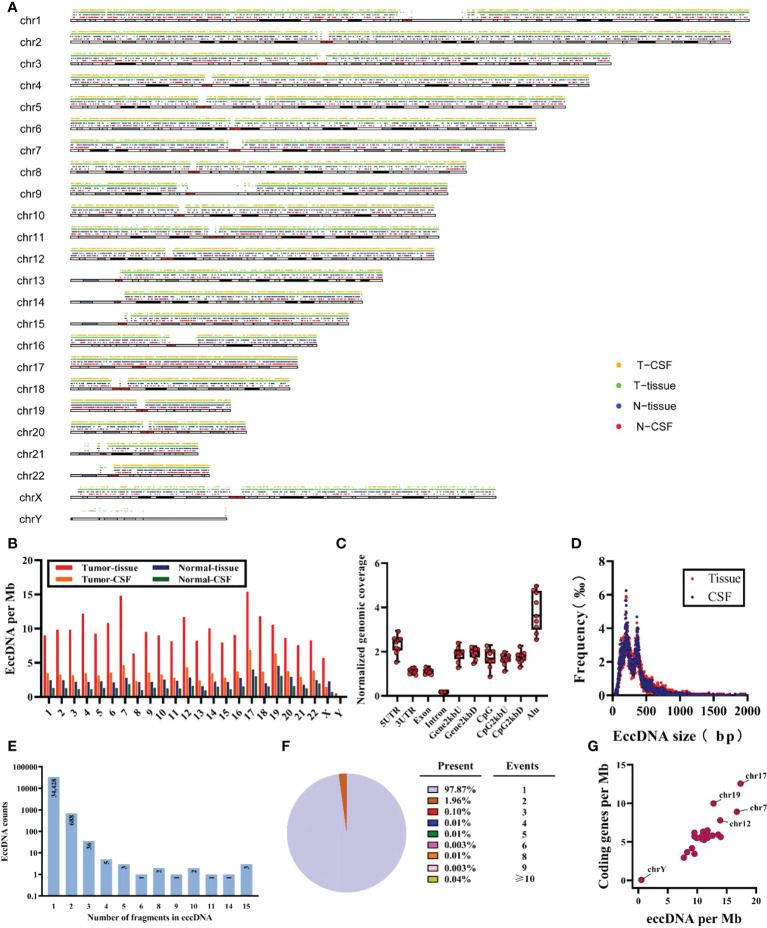
Characteristics of total eccDNAs. **(A)** Distribution for eccDNAs of four sample groups in chromosomes. **(B)** Frequency (per Mb) of distribution of eccDNAs in 23 pairs of chromosomes for each group. **(C)** The distribution of all samples of eccDNAs in different genomic regions. **(D)** Size distribution and relative abundance of eccDNAs in tissue (red) and CSF (blue), data from all samples. **(E, F)** The counts and proportion of eccDNAs in all samples cover different numbers of gene fragments. **(G)** The ratio of coding genes/Mb and eccDNAs/Mb in 23 pairs of chromosomes from analysis of all samples. Mb, megabase.

A total of 35,179 eccDNAs was detected in nine samples, containing 34,308 eccDNAs in tissue samples and 12,058 eccDNAs in CSF samples. These eccDNAs originated from all chromosomes; however, chromosome 17 exhibited the highest density of eccDNAs and was associated with more DNA damage-repair-related genes (**Figures 2A, B**) (36, 37). Interestingly, we found the least number of eccDNA on chromosome Y in normal tissue and CSF samples, consistent with the eccDNA profile reported in the previous literature (19). Localization of eccDNA to different component regions of the genome was conducted as previously described in the literature, defined as the percentage of eccDNA localized to that class of genomic regions divided by the percentage of the genome covered by that class of genomic regions (17). We found that eccDNAs were enriched in 5’-untranslated regions (5’UTRs) and Alu repeat regions, with the lowest distributions in the intronic regions (**Figure 2C**). The size of eccDNAs of all samples ranged from 32 to 7,239,203 bp, with 16 eccDNAs larger than 1 MB (0.4548‰), and most (35,098/35,179, 99.77%) eccDNAs were less than 2 kb with the median size of 272 and 279 bp in tissue and CSF, respectively (**Figure S3A**). Both tissue and CSF eccDNAs showed no variability in length distribution and exhibited two distinctive peaks at 201 bp and 360 bp (**Figure 2D**).

Meanwhile, after mapping all eccDNAs detected onto the whole genomic chromosomal region, 47.57% (16,733/35,179) of eccDNA overlapped with gene regions, of which 47.50% (16,297/34,308) and 50.07% (6,038/12,058) of eccDNAs covered gene fragments in tissue and CSF samples, respectively; 97.87% (34,428/35,179) of the detected eccDNAs were mapped to only one gene region. Unexpectedly, some could carry multiple gene fragments (**Figures 2E, F**); eight of all eccDNAs contained more than 15 gene fragments, which are not shown here considering that they may be due to chromosomal rearrangements. In addition, we found that 77.35% (13,943/18,026) of genes were present on more than two eccDNAs (**Figure S3B**), and the CNTNAP2 gene formed 43 unique eccDNAs, which may be related to the fact that CNTNAP2 encompasses almost 1.5% of chromosome 7 and is one of the largest genes in the human genome (38). Finally, as shown in **Figure 2G**, a positive correlation was found between eccDNAs/Mb and encoding genes/Mb, with a significantly higher average rate of eccDNA/Mb for chromosome 17 compared to other chromosomes.

### Differentially expressed eccDNAs between MB and normal tissues

**Figure 3:**
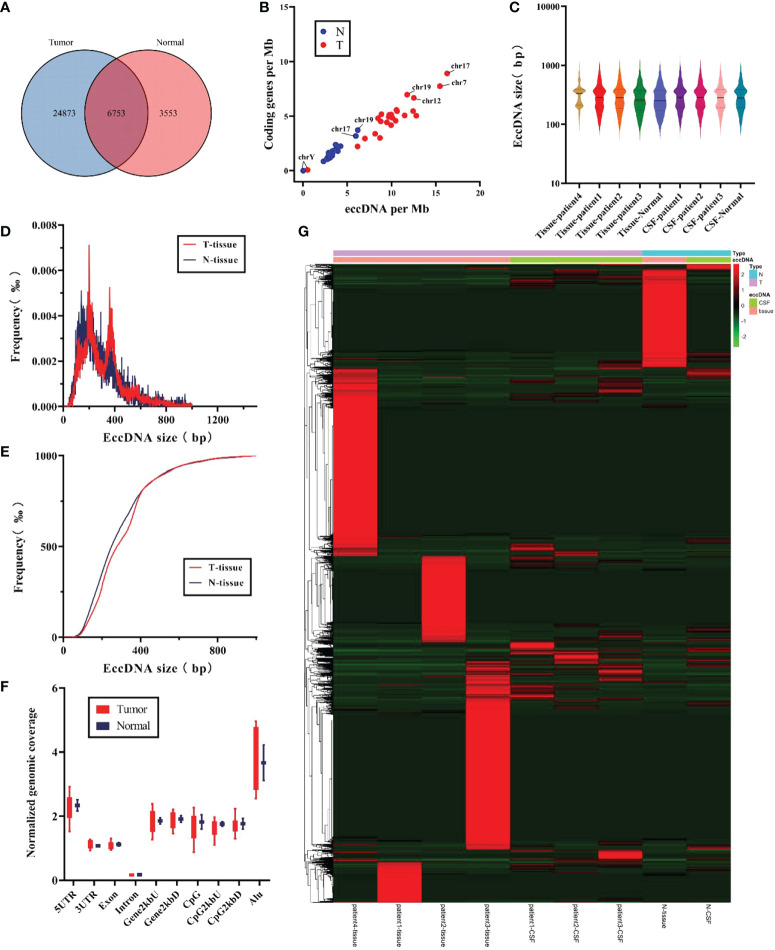
Comparison of the characteristics for eccDNAs in tumor samples and normal samples. **(A)** Differences in the counts for eccDNAs were detected in tumor and normal samples. **(B)** The ratio for coding genes/Mb and eccDNAs/Mb of chromosomes in tumor and normal samples. **(C)** Distribution of eccDNA length in each independent sample. **(D, E)** Size distribution of eccDNAs in tissue for tumor (red) and normal (blue). **(F)** Genomic distributions of eccDNAs between tumor (red) and normal (blue). **(G)** Heat map of abundance of eccDNAs in each sample.

Next, we compared whether there were differences in eccDNAs between tumor and normal samples. As shown in **Figure 3A**, 24,873 out of 35,179 eccDNAs were present only in all tumor samples, 3,553 were present in the normal samples, and 6,753 were detected in both samples. However, tumor and normal samples did not show differences between eccDNAs/Mb and encoding genes/Mb (**Figure 3B**). No significant variation in the length distribution of eccDNAs was found in each sample (**Figure 3C**). Interestingly, by comparing the length characteristics of eccDNAs of normal and tumor tissue samples, we found peaks of eccDNA at ∼201 and ∼306 bp in tumor tissues and 140 and 206 bp in normal tissues (**Figure 3D**). Subsequently, we analyzed the cumulative frequency to further explore the differences in their length characteristics (**Figure 3E**) and found that the length of normal tissue eccDNAs was smaller than tumor tissues. We compared the distribution of eccDNA length between tumor and normal CSF samples, and no significant differences were found (**Figures S4A, B**). Notably, we did not find significant differences in genomic elements of annotated eccDNAs between tumor and normal samples (**Figure 3F**).

Finally, the abundance of eccDNA in all samples was evaluated, and no significant difference was found (**Figure 3G**), probably due to the limited number of samples. However, analysis of the abundance of eccDNA in CSF samples showed that some eccDNAs were overexpressed in all three tumor CSF samples compared with normal samples (**Figure S4E**), suggesting that these eccDNAs have huge prospects for clinical application to distinguish tumors from the normal brain and may be involved in tumorigenesis and progression.

### EccDNAs between tissue and matched CSF in MB

**Figure 4:**
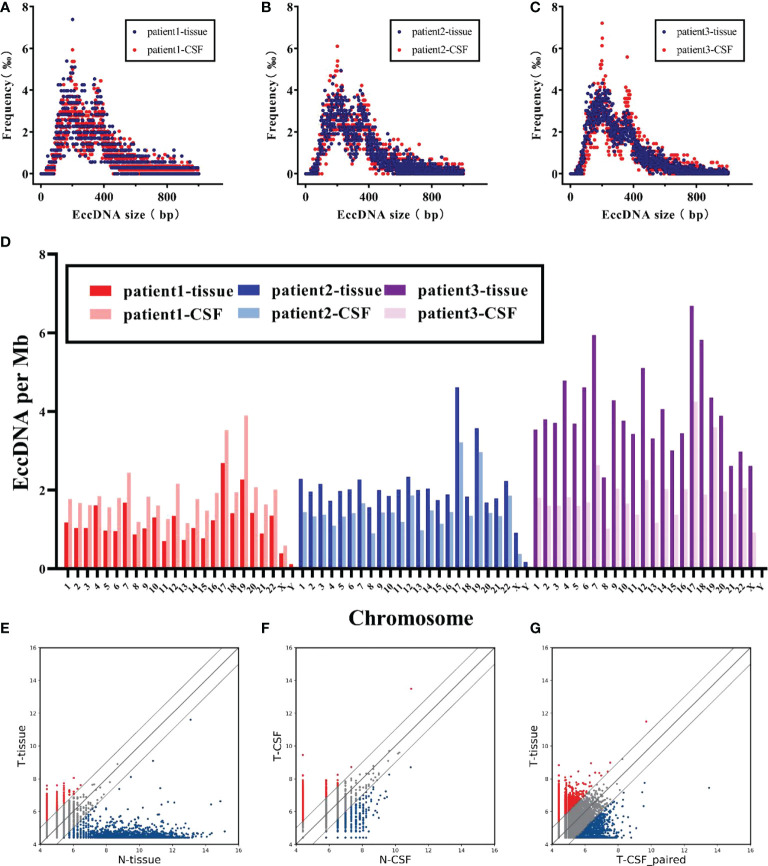
Characteristics of eccDNAs were comparable in MB and matched CSF. **(A–C)** Size distribution of eccDNAs in tissue (red) and CSF (blue) for each patient. **(D)** Comparison of chromosomal density trend of eccDNAs in tissue and CSF for three patients. **(E, F)** Scatter plots showing the differential abundance of eccDNAs between MB and normal in tissue and CSF, respectively. **(G)** Correlation of eccDNAs abundance between MB tumors and matched CSF.

To verify whether the states of eccDNAs in tumor tissue and matched CSF were consistent, we investigated the characteristics and abundance levels of eccDNA in three groups of patients, respectively. The eccDNA counts of tissue samples and matched CSF samples in the three groups of patients were as follows: group 1 (tissue: 3,508, CSF: 5,340), group 2 (tissue: 6,199, CSF: 4,221), and group 3 (tissue: 12,071, CSF: 5,471), with minimal interindividual differences in each group. As shown in **Figures 4A–C**, eccDNAs in both the tissue and matched CSF samples of the three groups did not exhibit significant differences in length distribution. On the other hand, tissue samples exhibited similar chromosome distribution with their matched CSF samples to a certain extent (**Figure 4D**). Finally, we compared the levels of eccDNA abundance between tumor and normal tissue samples, between tumor and normal CSF samples, and between tumor tissue samples and match CSF samples (**Figures 4E–G**). No significant difference in eccDNA abundance was found between tumor samples and matched CSF samples, suggesting that CSF has huge prospects to replace tumor samples as a means of detection and provides a new direction for monitoring eccDNA abundance levels in MB.

### Functional and pathways enrichment of the CSF differentially expressed eccDNAs

**Figure 5:**
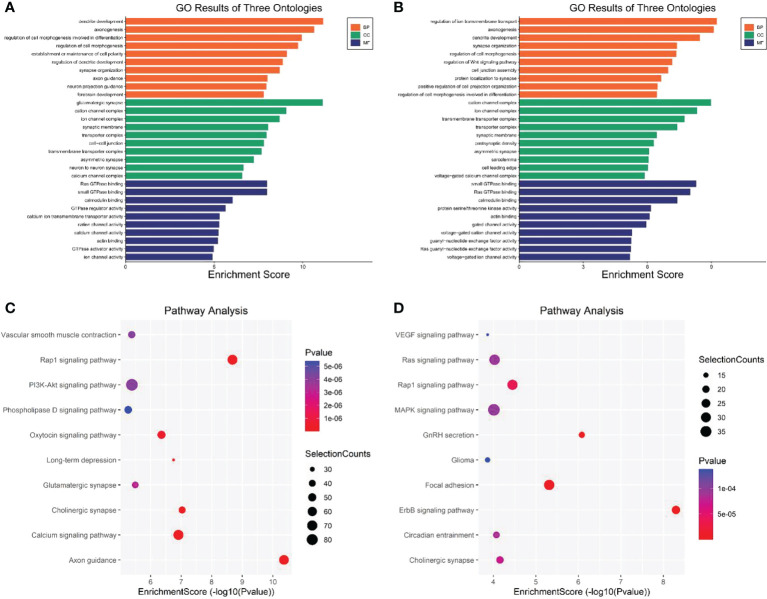
GO and KEGG pathway analysis of genes associated with the differentially expressed eccDNAs in CSF between MB and normal tissues. **(A)** Top 10 enriched BP, CC, and MF terms associated with the upregulated eccDNA genes. **(B)** Top 10 enriched BP, CC, and MF terms associated with the downregulated eccDNA genes. **(C, D)** KEGG pathway analysis of the upregulated and downregulated eccDNA genes, respectively. GO, Gene Ontology; KEGG, Kyoto Encyclopedia of Genes and Genomes; BP, biological processes; CC, cellular components, MF molecular functions (http://www.bioinformatics.com.cn/).

GO and KEGG pathway enrichment analyses were used to analyze the biological processes and functions of genes associated with the differentially expressed eccDNAs in CSF samples. As shown in **Figure 5A**, the top three enriched terms of the biological process associated with upregulated eccDNAs were “dendrite development”, “axonogenesis”, and “regulation of cell morphogenesis involved in differentiation”; the top three enriched terms of the cellular component were “glutamatergic synapse”, “cation channel complex”, and “ion channel complex”; the top three enriched terms of the molecular function were “Ras GTPase binding”, “small GTPase binding”, and “calmodulin binding”. Similarly, the top three enriched biological process terms associated with downregulated eccDNAs were “regulation of ion transmembrane transport”, “axonogenesis”, and “dendrite development”; the top three enriched cellular component terms were “cation channel complex”, “ion channel complex”, and “transmembrane transporter complex”; the top three enriched molecular function terms were “small GTPase binding”, “Ras GTPase binding”, and “calmodulin binding” (**Figure 5B**). In addition, KEGG pathway analysis showed that genes associated with differentially expressed eccDNA were significantly enriched in “Axon guidance pathway” (upregulated), “Rap1 signaling pathway” (upregulated), and “Cholinergic synapse pathway” (upregulated), and “ErbB signaling pathway” (downregulated), “GnRH secretion pathway” (downregulated), and “Focal adhesion pathway” (downregulated) (**Figures 5C, D**).

### Identification of survival-related hub genes associated with the differentially expressed eccDNAs in the CSF derived from normal and MB subjects

**Figure 6:**
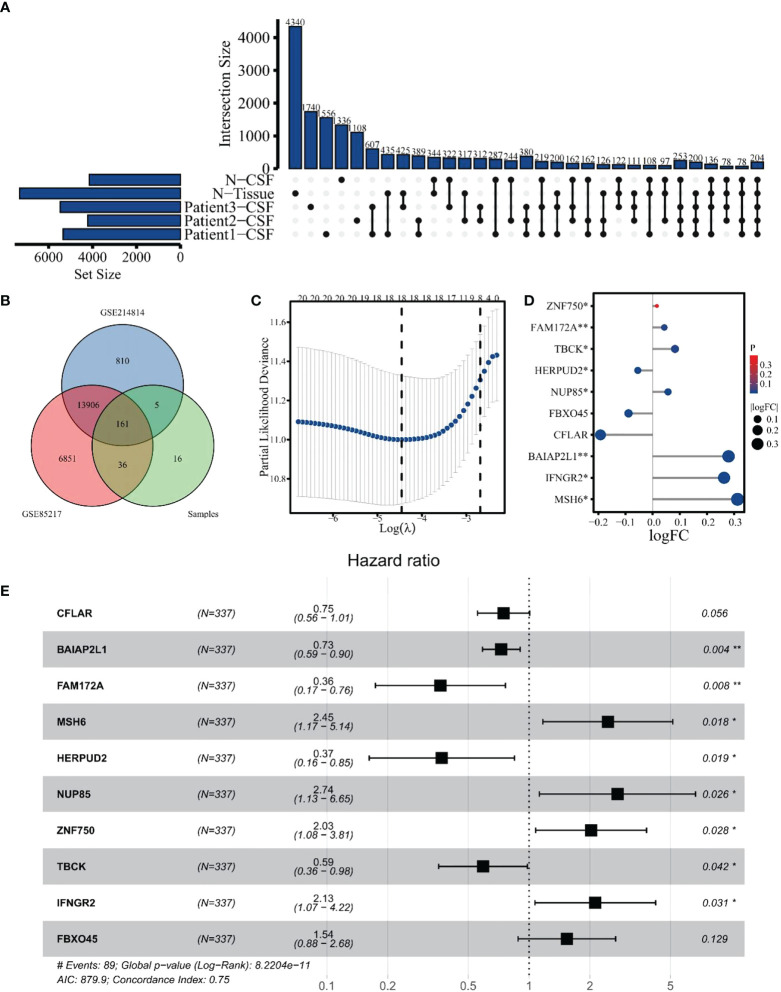
Identification of hub genes for differentially expressed eccDNAs in CSF. **(A)** A total of 380 differentially expressed eccDNAs between normal and MB in CSF. **(B)** Venn plot of genes among GSE124814, GSE85217, and data from our cohort. **(C)** Eighteen genes were selected by Lasso-penalized Cox analysis. **(D)** Differential expression of 10 hub genes between normal and tumor samples in dataset GSE124814. **(E)** Ten hub genes associated with OS in the training cohort. The symbol * means FDR adjusted p value for the labeled correlation was less than 0.05, ** means FDR adjusted p value for the labeled correlation was less than 0.01.

A total of 380 eccDNAs were expressed in three CSFs but not in normal samples. As previously mentioned, these eccDNAs contained 220 gene fragments, considered differential genes between the tumor and normal groups (**Figure 6A**). These genes were then intersected with genes in datasets GSE85217 and GSE124814, yielding 161 genes for subsequent analysis (**Figure 6B**). A total of 337 patients with clinical survival and follow-up information from the GEO dataset GSE85217 were included in the following survival analysis as the training cohort. Based on the univariate Cox regression model, 21 hub genes significantly correlated with the OS. Lasso-penalized Cox analysis identified 18 genes to be incorporated in multivariate Cox analysis (**Figure 6C**), and 10 genes were finally used to establish a prognostic model comprising MSH6, NUP85, TBCK, HERPUD2, ZNF750, BAIAP2L1, IFNGR2, FAM172A, FBXO45, and CFLAR. Interestingly, when we compared the expression of these hub genes with samples in the GSE214814 dataset (*n* = 1,641: 1,350 MB and 291 normal brain samples), 9 of these genes were differentially expressed (*p* < 0.05) (**Figure 6D**). Univariate Cox regression analysis demonstrated that these genes were independent prognostic factors of OS (*p* < 0.05), although the multivariate Cox regression analysis showed no significant association between FBXO45 and CFLAR and OS (**Table S6**). Finally, we included all 10 genes to establish the risk score model (**Figure 6E**).

### Establishment of the prognostic signature of hub genes

**Figure 7:**
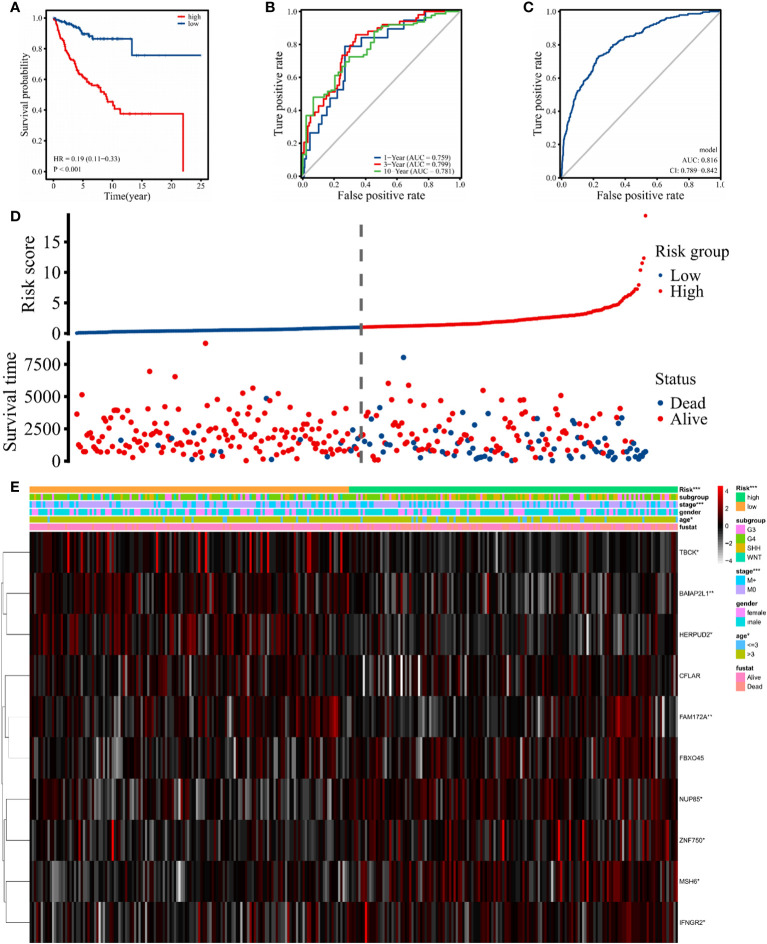
Signature-based risk score in the training cohort. **(A)** The Kaplan–Meier survival curves of the high (red) and low (blue) risk groups are based on the 10 hub gene signature. **(B)** The time-dependent ROC curves (1 year, 3 years, and 10 years) of the 10 hub gene signature. **(C)** ROC curve of the sensitivity for MB diagnostic through the 10 hub gene signatures. **(D, E)** Distribution of risk score, survival overview, and heatmap of hub genes in the training cohort. The symbol "*" means FDR adjusted p value for the labeled correlation was less than 0.05, "**" means FDR adjusted p value for the labeled correlation was less than 0.01, and "***" means FDR adjusted p value for the labeled correlation was less than 0.001.

The risk score was calculated based on the expression value of hub genes using the R package “survival”. Taking the median risk score as a cutoff value, 337 patients were assigned to high- or low-risk groups. The KM survival curve was plotted to compare OS between the two groups, and a significant difference was found (*p* < 0.001) (**Figure 7A**). The survival curves in **Figure S5A** demonstrate that the expressions of MSH6 (*p* = 0.028), NUP85 (*p* = 0.001), IFNGR2 (*p* = 0.017), and FBXO45 (*p* = 0.05) were negatively correlated with OS, and TBCK (*p* = 0.037), HERPUD2 (*p* = 0.001), and BAIAP2L1 (*p* = 0.008) were positively correlated. In addition, to assess the predictive power of the hub genes, time-dependent receiver operational characteristic (ROC) curves were used, yielding area under the curve (AUC) values of 0.759, 0.799, and 0.781 for 1-year, 3-year, and 10-year survival, respectively (**Figure 7B**). This finding suggests that these genes have a high sensitivity and specificity in predicting OS. Meanwhile, we found that the 3- and 10-year AUCs were higher than the 1-year AUC, indicating the stronger predictive power of hub genes for long-term outcomes. Moreover, ROC curve analysis showed that the AUCs of the intersected hub genes were >80% (**Figure 7C**), suggesting that they have a significant diagnostic value for MB. The individual ROCs for each gene are shown in **Figure S5B**, revealing the positive value of MSH6 and IFNGR2 as independent diagnostic factors. A comparison of the OS and the expression of 10 genes between the high-risk and low-risk groups showed that the high-risk group was associated with a poorer prognosis (**Figure 7D**). The expression of the upregulated genes correlated with worse patient prognosis (**Figure 7E**). As shown in **Figure S5C**, we established a clinically applicable nomogram for predicting the prognosis of MB patients based on the expression of these hub genes. All independent prognostic and associated gene expression parameters were included in the prognostic nomograms constructed by stepwise Cox regression models to predict 1-, 5-, and 10-year OS of MB patients in the training cohort.

### Construction and validation of the predictive nomogram

**Figure 8:**
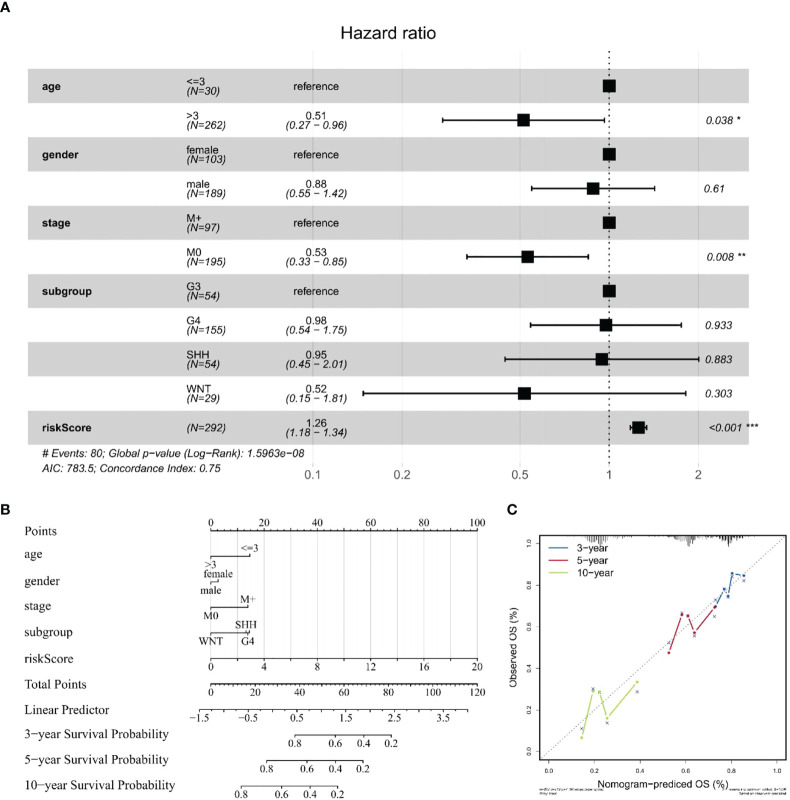
Construction and validation of a nomogram for survival prediction. **(A)** Multivariate Cox regression analysis of the association among clinical factors, risk score, and OS. **(B)** Nomogram combining the 10 hub gene signature and clinical factors. **(C)** Validation of the accuracy of the prognostic model with dataset GSE85217. The symbol "*" means FDR adjusted p value for the labeled correlation was less than 0.05, "**" means FDR adjusted p value for the labeled correlation was less than 0.01, and "***" means FDR adjusted p value for the labeled correlation was less than 0.001.

After verifying that the risk score could be used as an independent factor to predict OS of MB patients (*p* < 0.001), age, gender, tumor metastasis, and molecular subtype were included in our prognostic model (**Table S7**). Univariate and multivariate Cox regression analysis suggested that risk score (*p* < 0.001), age (*p* < 0.05), and tumor metastasis (*p* < 0.01) were all independent prognostic factors of OS in MB patients, unlike gender and molecular subtype (**Figure 8A**), consistent with the literature. Next, we established a nomogram to predict the OS of this patient population. The prognostic nomogram was constructed by a stepwise Cox regression model that included risk score, age, tumor metastasis, prognostic parameters, and relevant clinical data, to predict 3-, 5-, and 10-year OS of MB patients in the training cohort (**Figure 8B**). Finally, we compared the nomogram-predicted 3-, 5-, and 10-year OS with the observed 3-, 5-, and 10-year OS to validate the accuracy of the prognostic model, and both were generally consistent (**Figure 8C**), highlighting the reliability of our nomogram to predict the survival probability of MB patients.

## Discussion

Several studies suggest the presence of eccDNA in human plasma and tissues (17, 19, 39). Here, we extracted eccDNA from MB tumor tissue and matched CSF using two different methods based on Circle-Seq (17, 19, 35), substantiating the existence of eccDNA in CSF. It has been shown that eccDNA is closely related to the development of CNS tumors and affects the prognosis (10, 11, 16). An increasing body of evidence suggests that the genetic characteristics of cell-free tumor DNA (ctDNA) in CSF are consistent with matched CNS tumors and could be a reliable approach for monitoring the status of tumors (40–43). In the present study, we hypothesized that eccDNA might exhibit the genetic characteristics of MB tumors, and detecting the genetic characteristics of eccDNAs in CSF may provide a new direction for clinical diagnosis, treatment, and prognosis.

Importantly, the eccDNAs revealed in this study can be derived from all genomes and are roughly proportional to the overall abundance of non-repetitive and repetitive sequences in the genome, consistent with the literature (44, 45). Interestingly, the highest density of eccDNA distribution was found on chromosome 17, which may be because human autosomes have the highest gene density on chromosome 17 (46). The least amount of eccDNA was observed on chromosome Y, which has a low gene density. Consistently, when we mapped eccDNA to different classes of genomic elements to investigate their formation preferences, eccDNA was most enriched in the 5’UTR and the Alu repeat regions, which have the highest gene densities (47, 48). The pattern identified here was slightly different from that previously reported in the literature for eccDNAs in mice (49), human plasma (17), and esophageal squamous cell carcinoma (ESCC) (50), possibly due to the unique intracranial environment, although the exact reason remains to be further investigated. This distribution characteristic of eccDNA suggests that its formation may be inextricably linked to high gene density. To further search for the potential mechanism of eccDNA formation, we performed motif analysis of the nucleotide patterns around the breakpoints on both sides of eccDNAs. It has been suggested that double-repeat trinucleotide sequences on both sides of the breakpoints may be associated with eccDNA formation (17, 51), with increasing reports highlighting the presence of microhomologous base patterns at the junctions of eccDNAs, which can be formed by homologous recombination, microhomology end joining, or nonhomologous end joining-mediated cyclization of DNA (17, 18, 50, 52). A large number of repetitive bases were also identified in our data; unfortunately, after comparison with the repetitive sequences reported in the literature, we did not find any prominent features to prove an association with eccDNA formation. The current method of eccDNA purification is mainly through exonuclease digestion after alkaline lysis to obtain the product (19); this presents a problem that a trace amount of endonuclease activity in the exonuclease can cause eccDNA loss and thereby affect the yield. Mann et al. demonstrated that current research methods on eccDNA may produce false positives in rolling circle amplification (RCA) (53), because of its dependence on many cycles of amplification, and susceptibility to template-switching artifacts. These factors contribute to the general limitations of eccDNA research. A recently published study describes a new three-step eccDNA purification (3SEP) procedure by adding a new step that allows eccDNA purification with high purity and reproducibility (39); however, further details of the comparison between the different methods deserve to be discovered in more studies.

Of 35,179 eccDNAs, 16,733 (47.57%) overlapped with regions encoding genes. Surprisingly, some genes corresponded to multiple eccDNAs. Identification of genes that could form more than 20 eccDNAs showed that they were larger than 1 Mb (**Table S8**), which may reveal a genetic preference for eccDNA formation.

Consistent with previous studies, most eccDNAs (99.77%) were smaller than 2 kb and exhibited two distinct peaks at 201 bp and 360 bp (19, 54). Comparison of the eccDNA characteristics between the normal and tumor groups showed that the length of normal tissue eccDNAs was smaller than that of tumor tissues, similar to the difference between fetal and maternal eccDNA in the plasma (17). Although we did not observe differences in chromosome distribution, genomic elements of annotated eccDNAs, and repetitive sequences between the tumor and normal groups, the two groups exhibited greater variability in eccDNA abundance level, a phenomenon previously reported in a study on ESCC (50). Surprisingly, the abundance levels of eccDNAs in tumor tissue samples and matched CSF samples were related, while tumor and normal brain tissue samples were significantly different. Furthermore, the morphological characteristics and genomic distribution of eccDNAs in tumor tissue samples and matched CSF samples were consistent, highlighting that eccDNA levels in the CSF of MB patients can reflect the status of eccDNA levels in MB, warranting further investigation. Interestingly, we found that some eccDNAs were expressed in all three tumor CSF samples, which were lowly expressed in normal samples, suggesting that these eccDNAs may be potential biomarkers in distinguishing tumor from normal brain tissue and may be involved in tumorigenesis, progression, and evolution. Although our findings are clinically positive, unfortunately, there were not enough matched normal samples to further validate the differences between the tumor and normal groups, and the samples did not cover all MB subtypes, leaving us unable to investigate the characteristics of eccDNA in each MB subtype, which requires more patient data to increase its robustness.

To understand the underlying mechanisms, GO and KEGG pathway analyses of differentially expressed eccDNA-related genes in tumor CSF samples and normal CSF samples were conducted and revealed enrichment in “dendrite development”, “axonogenesis”, “Axon guidance pathway”, and “Rap1 signaling pathway”, which have been associated with CNS tumors (55, 56).

MB is one of the most common malignant tumors of the CNS in children with a poor prognosis (1, 4, 5). After the new addition of molecular biological markers in the 2016 edition of the World Health Organization (WHO) CNS tumor classification (6), genetic testing plays an important role in the staging and treatment of MB, including CTNNB for the WNT group (57), TP53 for the SHH group (58), MYC or MYCN for group 3 (59), and methylation for group 4 (60); these mutations are strongly associated with poor prognosis in MB patients. The sensitivity of magnetic resonance imaging (MRI) and CSF cytology, currently used in the clinical setting for patients with MB, is limited by the extent of tumor growth (7, 8), and monitoring tumor status that may not be found by imaging techniques can help physicians diagnose and treat earlier, contributing to the OS of patients (61). Importantly, previous literature has shown that the abundance of eccDNA is closely correlated with gene expression profiles (10, 62); thus, we hypothesized that a set of eccDNA-related genes might exhibit better performance in predicting the prognosis of MB patients. Subsequently, we analyzed two mRNA microarray datasets (GSE85217 and GSE124814), combined with the expression data of eccDNA overlapping genes measured in our cohort samples. Univariate Cox analysis, Lasso Cox regression analysis, and multivariate Cox analysis were performed, and 10 hub genes were incorporated to construct the risk model comprising MSH6, NUP85, TBCK, HERPUD2, ZNF750, BAIAP2L1, IFNGR2, FAM172A, FBXO45, and CFLAR. Although these eccDNA-related genes expressed some correlation after comparing our data with GSE124814, which is consistent with previous studies (10, 62), the overall association between the two datasets was not significant due to the different sources. More work will be carried out to reveal the correlation between eccDNA and gene expression profiles in MB by including a larger sample in the future. A total of 337 patients with clinical survival and follow-up information from the GEO dataset GSE85217 were included in the following survival analysis. We evaluated the model’s performance using ROC curves of the risk score obtained from the combined analysis of hub gene expression. ROC curve analysis yielded AUCs of 0.759, 0.799, and 0.781 for survival at 1, 3, and 10 years, respectively, implying that the risk score had high sensitivity and specificity. We also assessed the diagnostic performance of the hub gene in MB patients. Finally, we constructed a nomogram to predict the OS of MB patients, which exhibited better performance than traditional clinical factors (gender, grading, age, etc.), and validated its accuracy and sensitivity using prognostic data from real patients. Compared to previous literature that identified a 12-gene signature (AUC of 1 year = 0.889, AUC of 3 years = 0.681, and AUC of 5 years = 0.703) to predict OS in MB by considering only the GEO database (63), our data (AUC of 1 year = 0.759, AUC of 3 years = 0.799, and AUC of 10 years = 0.781) show stronger long-term predictive power in prognosis, and one strength of eccDNA is the ability to profile it in the CSF. However, this model has not been further validated in experiments and was based on a relatively small sample size of data.

In addition, 7 of these 10 hub genes reportedly participate in the biological processes of the tumor. MSH6 is a protein-coding gene component of the post-replicative DNA mismatch repair system (MMR) and forms a heterodimer with MSH2 to form MutS α, which is involved in DNA repair. Growing evidence suggests that MSH6 expression is significantly associated with tumor drug resistance and poor clinical outcomes, especially in MB, glioblastoma, bladder cancer, and breast cancer (64–66). MSH6 also showed high accuracy and sensitivity in diagnosis and prognosis in our data analysis, highlighting that it is an area worthy of our focus. NUP85 is related to the composition of the Nup107–160 subunit of the nuclear pore complex, mostly associated with nephrotic syndrome (67). Recent studies have identified its possible involvement in tumor development through the immune system and its potential as a new therapeutic target (68). ZNF750 is mainly expressed in squamous epithelial cells, with a nuclear localization signal and a conserved C2H2 zinc finger domain, and has been reported to correlate with the prognosis of ESCC, colonic cancer, and nasopharyngeal carcinoma patients (69–71). BAIAP2L1 is a protein-coding gene belonging to the IRSp53 family, which acts as an insulin receptor (IR) adapter that activates the IR-irs1/2 (insulin receptor substrate 1/2)–AKT signaling pathway by stimulating tyrosine phosphorylation of IR (72). BAIAP2L1 has been reported as a potential biomarker in various tumors (73–75). FAM172A is a newly discovered protein-coding gene whose specific function has not been studied, although it is widely thought to regulate alternative splicing by interacting with AGO2 and CHD7 (76), especially in pancreatic cancer and papillary thyroid carcinoma (76, 77). FBOX45 belongs to the FBXO protein subfamily and has been closely associated with the development of the nervous system. Recent studies have shown that FBOX45 is also involved in cancer development (78, 79), but the exact mechanism has not yet been studied. Moreover, CFLAR (CASP8 And FADD Like Apoptosis Regulator) plays an important role in several cellular processes such as apoptosis, necrosis, autophagy, and inflammation and is structurally similar to caspase-8 (80). An increasing body of evidence from recently published studies suggests that CFLAR overexpression contributes to tumor progression and correlates with a poor clinical outcome in cancers such as prostate, colorectal, gastric cancers, head and neck squamous cell carcinoma (HNSCC), and non-small cell lung cancer, which may be related to the cell death inhibitory function of FLIP (81, 82). Moreover, the protein encoded by HERPUD2 may be involved in the endoplasmic reticulum (ER)-associated degradation and mediates ER stress-induced inflammation (83). Furthermore, TBCK (TBC1 domain containing kinase) may be involved in the transcriptional regulation of components of the mammalian target of the rapamycin (mTOR) complex and has also been associated with neuronal developmental disorders (84, 85). Finally, IFNGR2 (interferon-gamma receptor 2) encodes a protein that is the non-ligand binding β-chain of the gamma interferon receptor, and its possible involvement in interleukin (IL)-1β-dependent inflammation was noted in recent studies (86, 87).

Notwithstanding that several articles have studied the potential functional mechanisms of eccDNAs in various tumors by combining next-generation sequencing, few have explored the association between eccDNAs and MB. Herein, we demonstrated the presence of eccDNAs in CSF, described the characteristics and genomic landscape of eccDNAs, and researched the possible mechanisms of its production. EccDNAs exhibit similarities between MB and matched CSF, suggesting their potential as a biomarker in the diagnosis and prognosis of MB. Based on the differentially expressed eccDNA-related genes between tumor and normal CSF samples, combined with the GEO database analysis, we screened 10 hub genes associated with MB diagnosis and prognosis and established a model to benefit patients and physicians in clinical practice. Our findings also reveal the potential of eccDNA in targeted interventions. However, limited by the relatively small clinical sample size, individual variations in eccDNAs among the samples from this study may affect the robustness of our findings to a certain extent, emphasizing the need for more studies with larger sample sizes.

## Data availability statement

The data presented in the study are deposited in the Gene Expression Omnibus (https://www.ncbi.nlm.nih.gov/geo/) repository, accession number GSE205178.

## Ethics statement

The studies involving human participants were reviewed and approved by Department of Neurosurgery, Sanbo Brain Hospital, Capital Medical University. Written informed consent to participate in this study was provided by the participants’ legal guardian/next of kin. Written informed consent was obtained from the individual(s), and minor(s)’ legal guardian/next of kin, for the publication of any potentially identifiable images or data included in this article.

## Author contributions

All authors contributed to the study conception, design, and writing. All authors have given approval for the final version of the manuscript.

## Funding

This work was supported by the National Key R&D Program (No. 2019YFC1316104), the National Science Foundation of China (No. 22077120), the China Postdoctoral Science Foundation (No. 2019M660715), and the independent project of Sanbo Brain Hospital (No. 2022ZZLX01).

## Conflict of interest

The authors declare that the research was conducted in the absence of any commercial or financial relationships that could be construed as a potential conflict of interest.

## Publisher’s note

All claims expressed in this article are solely those of the authors and do not necessarily represent those of their affiliated organizations, or those of the publisher, the editors and the reviewers. Any product that may be evaluated in this article, or claim that may be made by its manufacturer, is not guaranteed or endorsed by the publisher.
