# Supplementary material for: Whole-genome sequencing of extrachromosomal circular DNA of cerebrospinal fluid of medulloblastoma
Source: Front Oncol. 2022 Nov 8;12:934159. doi: 10.3389/fonc.2022.934159 (PMC9703567; doi:10.3389/fonc.2022.934159)
Supplement: Supplementary file 1 [file DataSheet_1.docx]

# Supporting Information

**Whole-genome sequencing of Extrachromosomal circular DNA in cerebrospinal fluid of Medulloblastoma**

Yi Zhu^1,2#^, Zhihui Liu^3#^, Yuduo Guo^4^, Shenglun Li^1^, Yanming Qu^1^, Lin Dai^1,2^, Yujia Chen^1^, Weihai Ning^1^, Hongwei Zhang^1^, Lixin Ma^1,2*^

1.Department of Neurosurgery, Sanbo Brain Hospital, Capital Medical University, Beijing, 100093, P. R. China

2.Department of Neurosurgery, Binzhou Medical University Hospital, Binzhou 256603, China

3.Department of Obstetrics and Gynecology, Beijing Chaoyang Hospital, Capital Medical University, Beijing, P. R. China

4. CAS Key Laboratory of Infection and Immunity, Institute of biophysics, Chinese Academy of Sciences, No 15 Datun Road, Chaoyang District, Beijing, PR China.

# Yi Zhu and Zhihui Liu contributed equally and should be considered first authors.

**Disclosure of Potential Conflicts of Interest:** The authors declare that no competing interests exist.

## Table S1. DNA Quantification by Qubit 3.0 Fluorometer

| **Sample Name** | **Sample ID** | **Conc.**  **(ng/μl))** | **Volume**  **(μl)** | **Quantity**  **(μg)** | **QC Results** |
| --- | --- | --- | --- | --- | --- |
| T-CSF- patient1 | 1 | 1.856 | 13 | 24.13 | Pass |
| T-CSF- patient2 | 2 | 1.970 | 13 | 25.61 | Pass |
| T-CSF- patient3 | 3 | 1.600 | 13 | 20.80 | Pass |
| N-CSF | 4 | 1.560 | 13 | 20.28 | Pass |
| T-tissue-patient4 | 5 | 260.000 | 43 | 11180.00 | Pass |
| T-tissue-patient1 | 6 | 253.750 | 43 | 10911.25 | Pass |
| T-tissue-patient2 | 7 | 290.000 | 43 | 12470.00 | Pass |
| T-tissue-patient3 | 8 | 236.000 | 43 | 10148.00 | Pass |
| N-tissue | 9 | 210.700 | 43 | 9060.10 | Pass |

## Table S2. Quality Assessment of Sequencing Library

| **Sample** | **Size**  **(bp)** | **Conc.**  **(ng/μl))** | **Conc.**  **(nmol/L))** | **Volume**  **(μl)** | **TotalAmount**  **(ng)** |
| --- | --- | --- | --- | --- | --- |
| T-tissue-patient4 | 316 | 1.44 | 7.00 | 10 | 14.4 |
| T-tissue-patient1 | 321 | 1.39 | 6.65 | 10 | 13.9 |
| T-tissue-patient2 | 324 | 2.19 | 10.4 | 10 | 21.9 |
| T-tissue-patient3 | 317 | 2.22 | 10.8 | 10 | 22.2 |
| T-CSF- patient1 | 301 | 2.13 | 10.9 | 10 | 21.3 |
| T-CSF- patient2 | 304 | 2.31 | 11.7 | 10 | 23.1 |
| T-CSF- patient3 | 304 | 1.73 | 8.72 | 10 | 17.3 |
| N-CSF | 293 | 1.70 | 8.95 | 10 | 17.0 |
| N-tissue | 337 | 1.73 | 7.89 | 10 | 17.3 |

## Table S3. Quantification of COX5B expression by qPCR

| **Sample** | **Pre-nuclease** | | | | | **Post-exonuclease** | | | | |
| --- | --- | --- | --- | --- | --- | --- | --- | --- | --- | --- |
|  | **Ct** | | | **Ct Mean** | **Ct SD** | **Ct** | | | **Ct Mean** | **Ct SD** |
| T-tissue-patient4 | 23.13 | 22.89 | 22.97 | 22.99 | 0.1215 | 30.19 | 30.26 | 30.27 | 30.24 | 0.0393 |
| T-tissue-patient1 | 22.62 | 22.57 | 22.71 | 22.63 | 0.0724 | 29.36 | 29.37 | 29.37 | 29.37 | 0.0036 |
| T-tissue-patient2 | 23.40 | 23.52 | 23.44 | 23.45 | 0.0632 | 29.46 | 29.49 | 29.52 | 29.49 | 0.0308 |
| T-tissue-patient3 | 23.83 | 24.11 | 24.08 | 24.01 | 0.1519 | 29.74 | 29.76 | 29.77 | 29.76 | 0.0138 |
| T-CSF- patient1 | 23.18 | 23.16 | 23.21 | 23.18 | 0.0253 | 29.23 | 29.33 | 29.35 | 29.30 | 0.0630 |
| T-CSF- patient2 | 23.58 | 23.71 | 23.66 | 23.65 | 0.0663 | 29.39 | 29.41 | 29.44 | 29.41 | 0.0266 |
| T-CSF- patient3 | 24.12 | 24.34 | 24.24 | 24.23 | 0.1127 | 29.55 | 29.57 | 29.57 | 29.56 | 0.0098 |
| N-CSF | 24.80 | 24.78 | 24.84 | 24.81 | 0.0303 | 29.89 | 29.89 | 29.93 | 29.91 | 0.0243 |
| N-tissue | 24.73 | 24.71 | 24.69 | 24.71 | 0.0158 | 30.01 | 30.04 | 30.14 | 30.06 | 0.0651 |

## Table S4. Q30 quality control for sample

| **Sample** | **Q30** |
| --- | --- |
| T-tissue-patient4 | 88.74% |
| T-tissue-patient1 | 85.85% |
| T-tissue-patient2 | 89.66% |
| T-tissue-patient3 | 89.37% |
| T-CSF- patient1 | 91.81% |
| T-CSF- patient2 | 91.08% |
| T-CSF- patient3 | 91.85% |
| N-CSF | 91.81% |
| N-tissue | 89.19% |

## Table S5. Statistics of reads for sample

| **Sample** | **Raw Reads** | **Clean Reads** | **Clean rate** | **eccDNA Count** |
| --- | --- | --- | --- | --- |
| T-tissue-patient4 | 149,588,850 | 149,579,718 | 99.9939% | 12178 |
| T-tissue-patient1 | 126,667,164 | 126,659,660 | 99.9941% | 3508 |
| T-tissue-patient2 | 143,472,382 | 143,464,198 | 99.9943% | 6199 |
| T-tissue-patient3 | 115,447,326 | 115,441,120 | 99.9946% | 12071 |
| T-CSF- patient1 | 147,075,806 | 147,063,950 | 99.9919% | 5340 |
| T-CSF- patient2 | 147,966,044 | 147,954,498 | 99.9922% | 4221 |
| T-CSF- patient3 | 181,415,186 | 181,403,600 | 99.9936% | 5471 |
| N-CSF | 170,568,216 | 170,557,662 | 99.9938% | 4153 |
| N-tissue | 147,977,682 | 147,968,692 | 99.9939% | 7321 |

## Table S6. Genes associated with OS

| **Variables** | **Univariate analysis** | | | | **Multivariate analysis** | | | |
| --- | --- | --- | --- | --- | --- | --- | --- | --- |
|  | **HR** | **HR.95L** | **HR.95H** | **P-value** | **HR** | **HR.95L** | **HR.95H** | **P-value** |
| BAIAP2L1 | 0.760706 | 0.612971 | 0.944047 | 0.013043 | 0.729297 | 0.588509 | 0.903766 | 0.003919 |
| FAM172A | 0.439797 | 0.226671 | 0.853312 | 0.015139 | 0.364547 | 0.17383 | 0.764511 | 0.007571 |
| MSH6 | 3.692888 | 1.872098 | 7.284568 | 0.000164 | 2.451409 | 1.169553 | 5.138207 | 0.01756 |
| HERPUD2 | 0.322547 | 0.158187 | 0.657682 | 0.001854 | 0.370362 | 0.1619 | 0.847237 | 0.018642 |
| NUP85 | 3.930487 | 1.787417 | 8.643051 | 0.000663 | 2.736065 | 1.125066 | 6.653878 | 0.026428 |
| ZNF750 | 1.952894 | 1.203775 | 3.168194 | 0.006703 | 2.026077 | 1.07741 | 3.810052 | 0.028426 |
| IFNGR2 | 2.168998 | 1.166168 | 4.034195 | 0.014465 | 2.125212 | 1.069291 | 4.223852 | 0.031466 |
| TBCK | 0.468799 | 0.292019 | 0.752597 | 0.001708 | 0.592225 | 0.357127 | 0.982089 | 0.042357 |
| CFLAR | 0.746851 | 0.558304 | 0.999072 | 0.049274 | 0.748361 | 0.55609 | 1.00711 | 0.055722 |
| FBXO45 | 1.649353 | 1.013819 | 2.683285 | 0.04388 | 1.537584 | 0.882434 | 2.67914 | 0.128887 |

## Table S7. Cox regression analysis of the association between clinical factors and OS.

| **Variables** | **Univariate analysis** | | | | **Multivariate analysis** | | | |
| --- | --- | --- | --- | --- | --- | --- | --- | --- |
|  | **HR** | **HR.95L** | **HR.95H** | **P-value** | **HR** | **HR.95L** | **HR.95H** | **P-value** |
| age(>3) | 0.494932 | 0.272365 | 0.899374 | 0.021 | 0.514025 | 0.2741 | 0.963958 | 0.038044 |
| gender(male) | 1.082044 | 0.680302 | 1.721028 | 0.739116 | 0.883145 | 0.547524 | 1.424494 | 0.61044 |
| stage(M0) | 0.56058 | 0.359995 | 0.872928 | 0.010425 | 0.530603 | 0.331134 | 0.850227 | 0.008428 |
| subgroup(G4) | 0.809548 | 0.459731 | 1.425547 | 0.464267 | 0.975226 | 0.543073 | 1.751266 | 0.933067 |
| subgroup(SHH) | 1.232524 | 0.63271 | 2.400965 | 0.53888 | 0.945046 | 0.445087 | 2.0066 | 0.883033 |
| subgroup(WNT) | 0.299104 | 0.087629 | 1.02093 | 0.053993 | 0.517872 | 0.147915 | 1.813151 | 0.303376 |
| riskScore | 1.240428 | 1.178295 | 1.305837 | 2.08E-16 | 1.255556 | 1.178857 | 1.337244 | 1.48E-12 |

## Table S8. Size of Genes correspond to multiple eccDNA

| **Genename** | | **Number of eccDNA generated** | **Size(bp)** | **Genename** | **Number of eccDNA generated** | **Size(bp)** |
| --- | --- | --- | --- | --- | --- | --- |
| CNTNAP2 | 43 | 2,304,997 | NRXN3 | 22 | 1,888,388 |  |
| DLG2 | | 31 | 2,173,527 | NAALADL2 | 22 | 1,372,015 |
| RBFOX1 | | 27 | 2,473,592 | MAGI2 | 21 | 1,436,613 |
| PDE4D | | 27 | 1,555,292 | MACROD2 | 21 | 2,057,829 |
| EYS | | 27 | 1,987,247 | DMD | 21 | 2,241,933 |
| AUTS2 | | 27 | 1,195,032 | CSMD1 | 21 | 2,059,683 |
| ASIC2 | | 26 | 1,161,878 | PCDH15 | 20 | 1,826,412 |
| DAB1 | | 24 | 1,551,957 | ANKS1B | 20 | 1,258,480 |


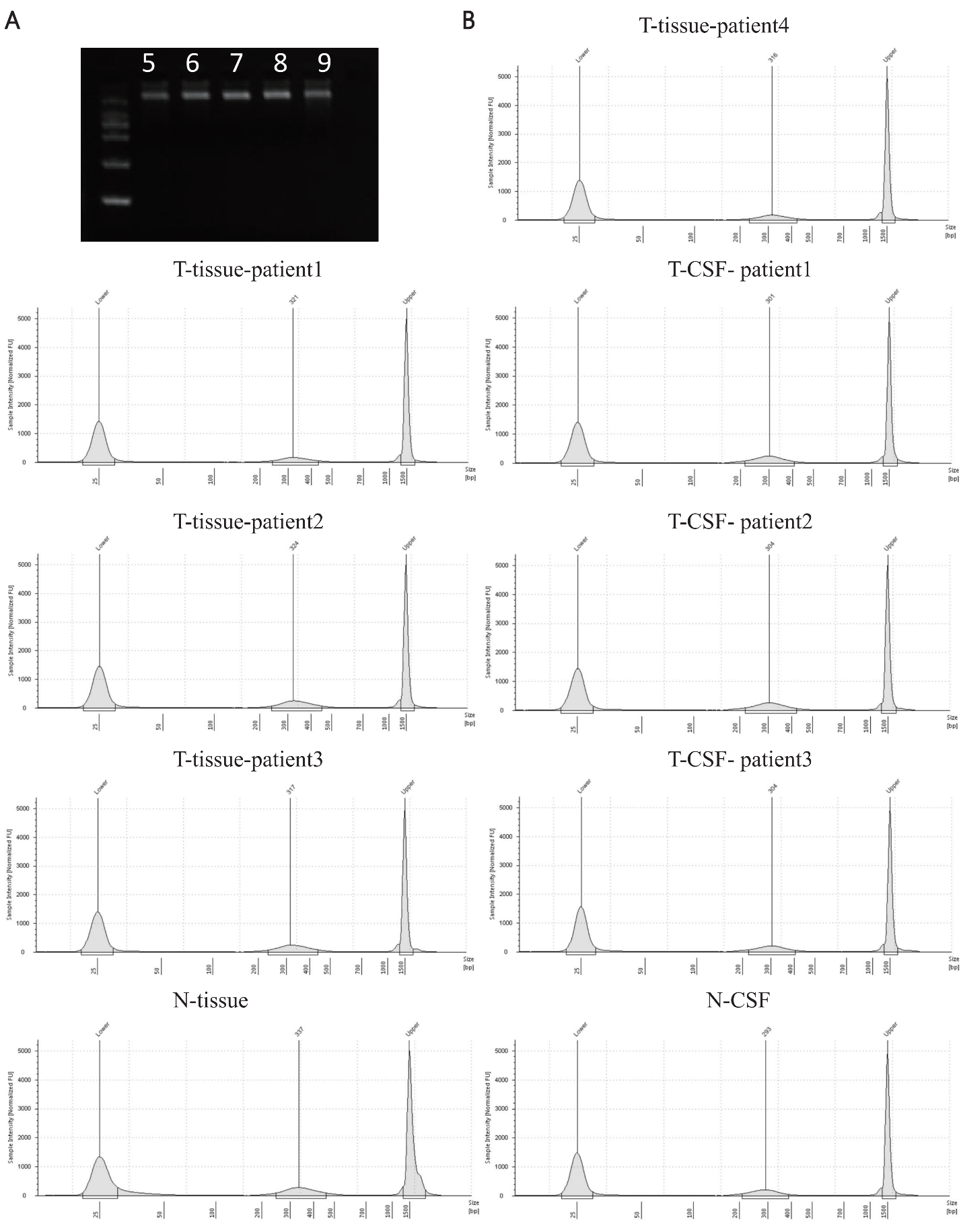
**Figure S1**. (A) DNA Integrity by Agarose Gel Electrophoresis. (B) Quality Assessment of Sequencing Library.


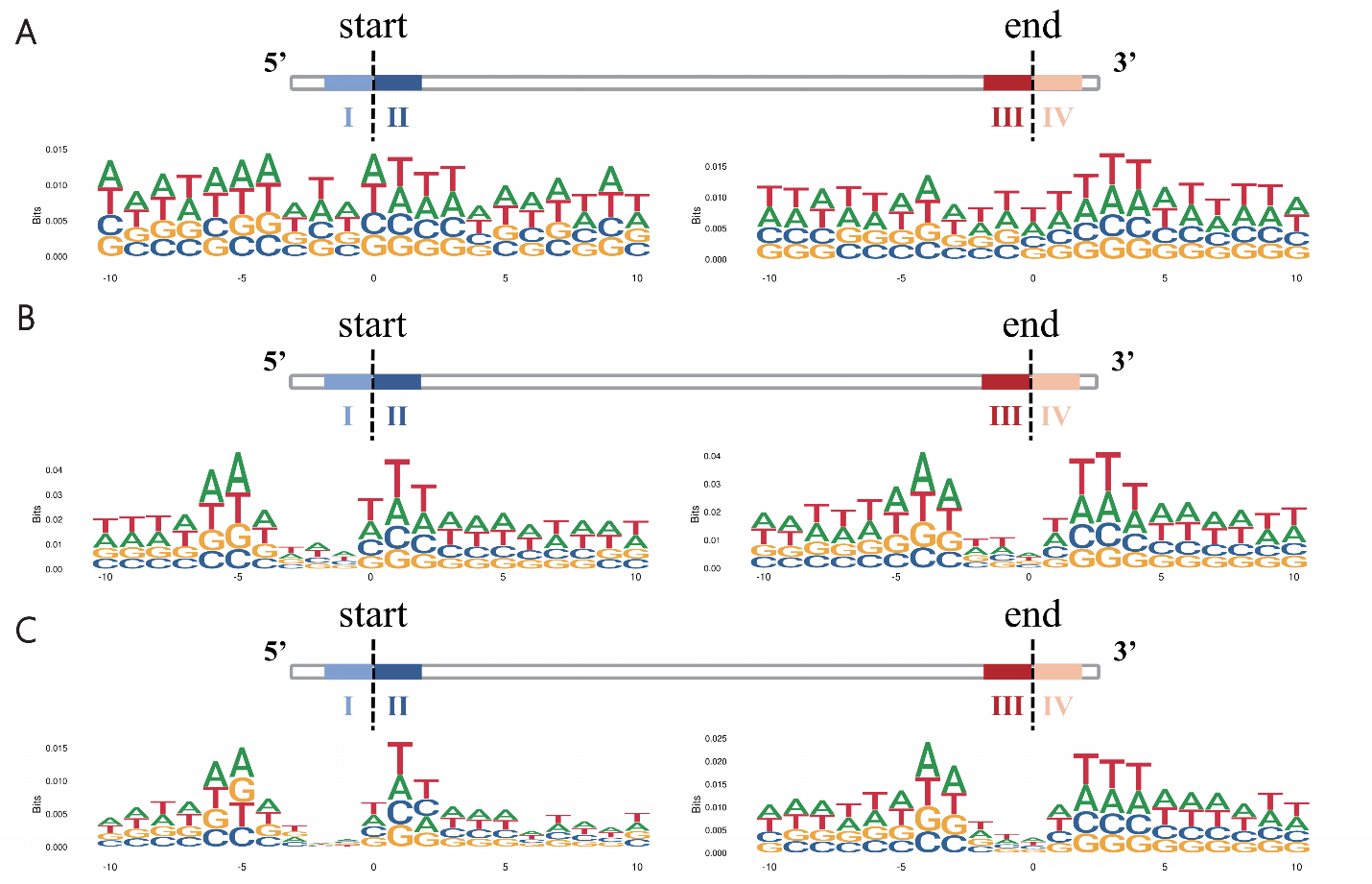


**Figure S2**. (A) Trinucleotide motif sequences flanking the start and end positions of eccDNA in normal tissue. (B) Trinucleotide motif sequences flanking the start and end positions of eccDNA in tumor tissue. (C) Trinucleotide motif sequences flanking the start and end positions of eccDNA in tumor CSF. eccDNAs, extrachromosomal circular DNAs; CSF, cerebrospinal fluid.


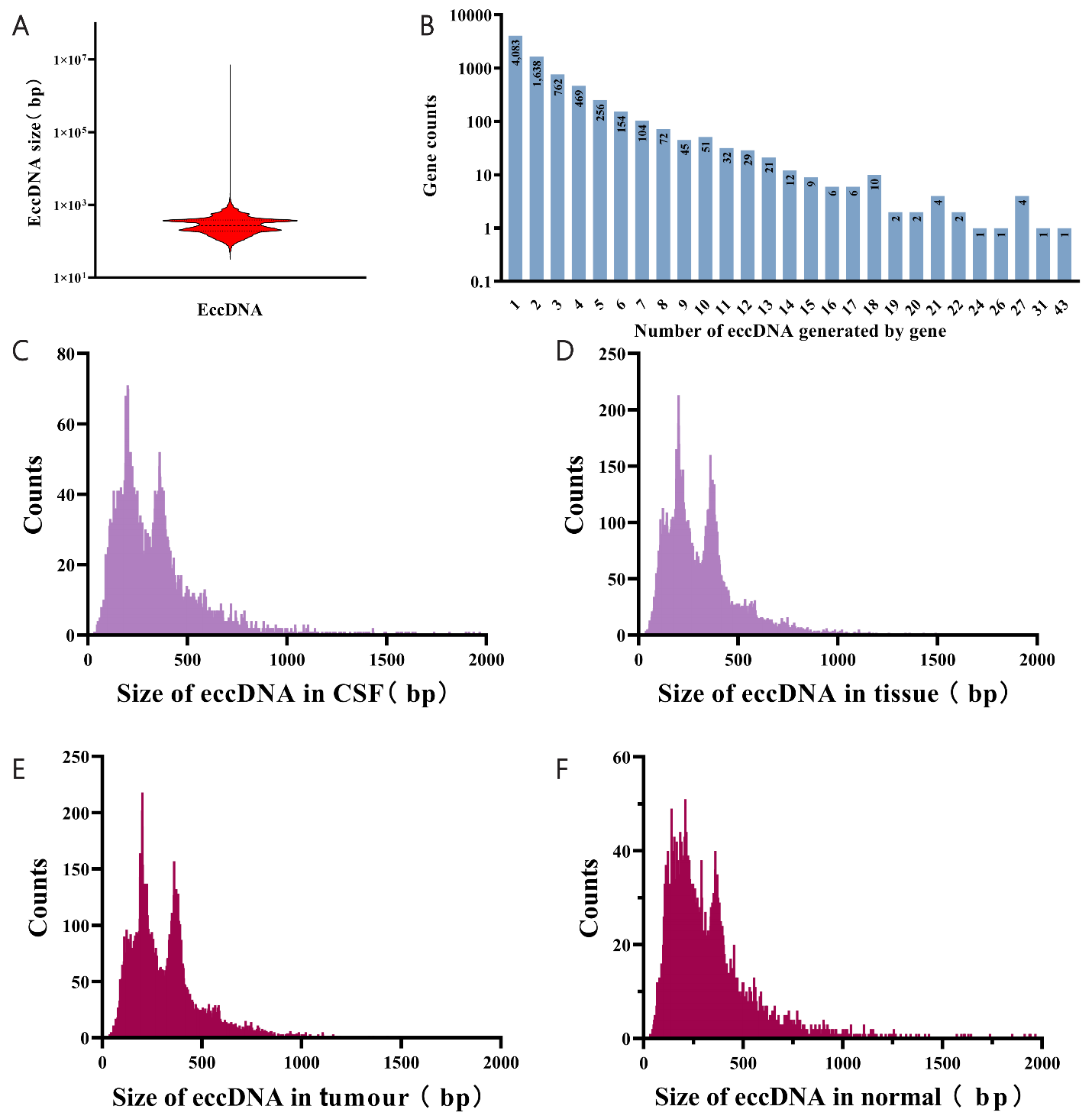


**Figure S3**. (A) Overall length distribution of eccDNAs. (B) Counts of Genes correspond to multiple eccDNA. (C-D) Comparison of eccDNA length distribution in CSF samples and tissue samples. (E-F) Comparison of eccDNA length distribution in tumor samples and normal samples.


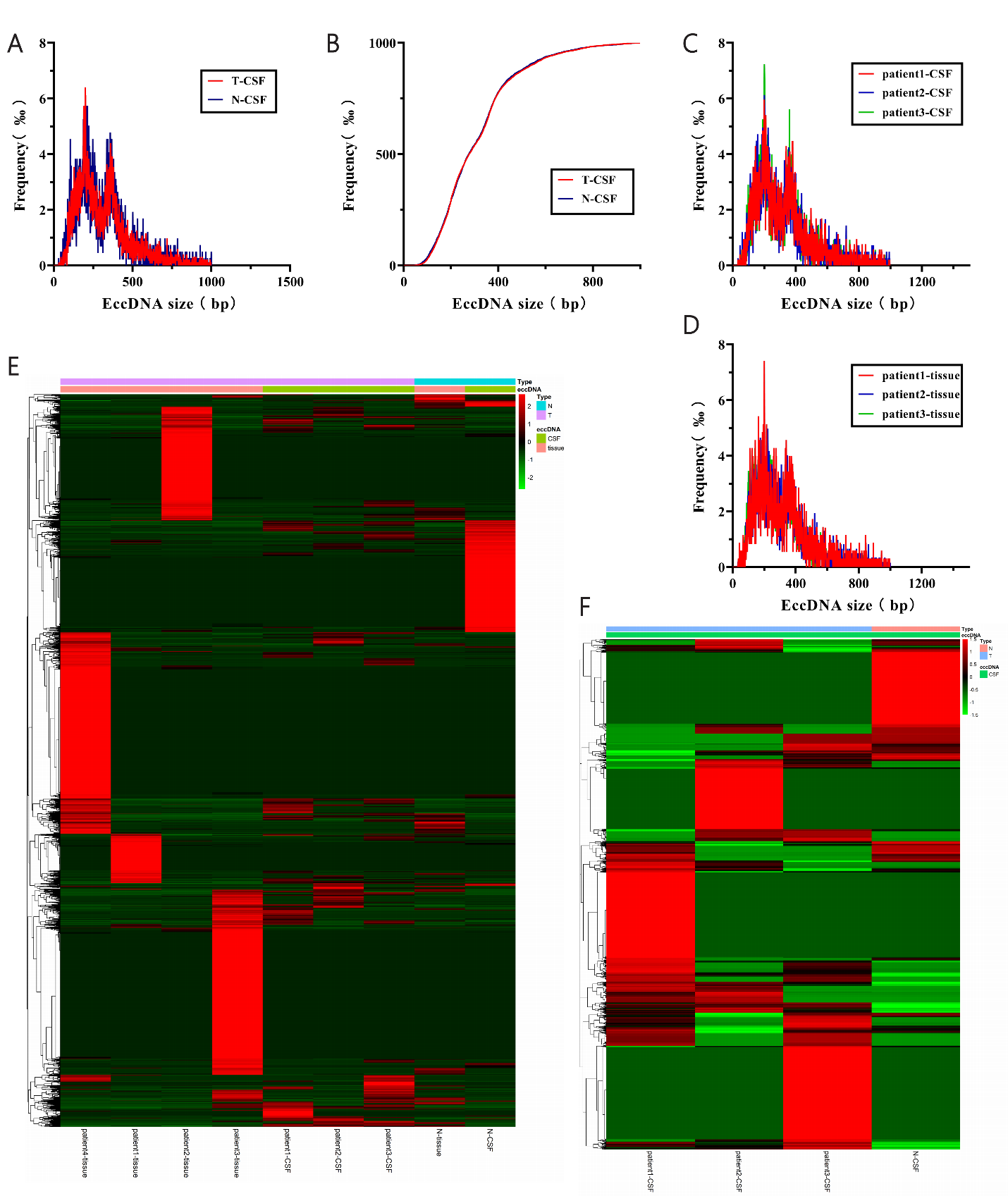


**Figure S4**. (A-B) Differences in eccDNAs length between tumor CSF samples and normal CSF samples. (C-D) Differences in length of eccDNA among patients in tumor tissue samples and CSF samples. (E) Heat map of expression of eccDNA in samples covering gene regions. (F) Heat map of eccDNA expression in CSF samples.

**
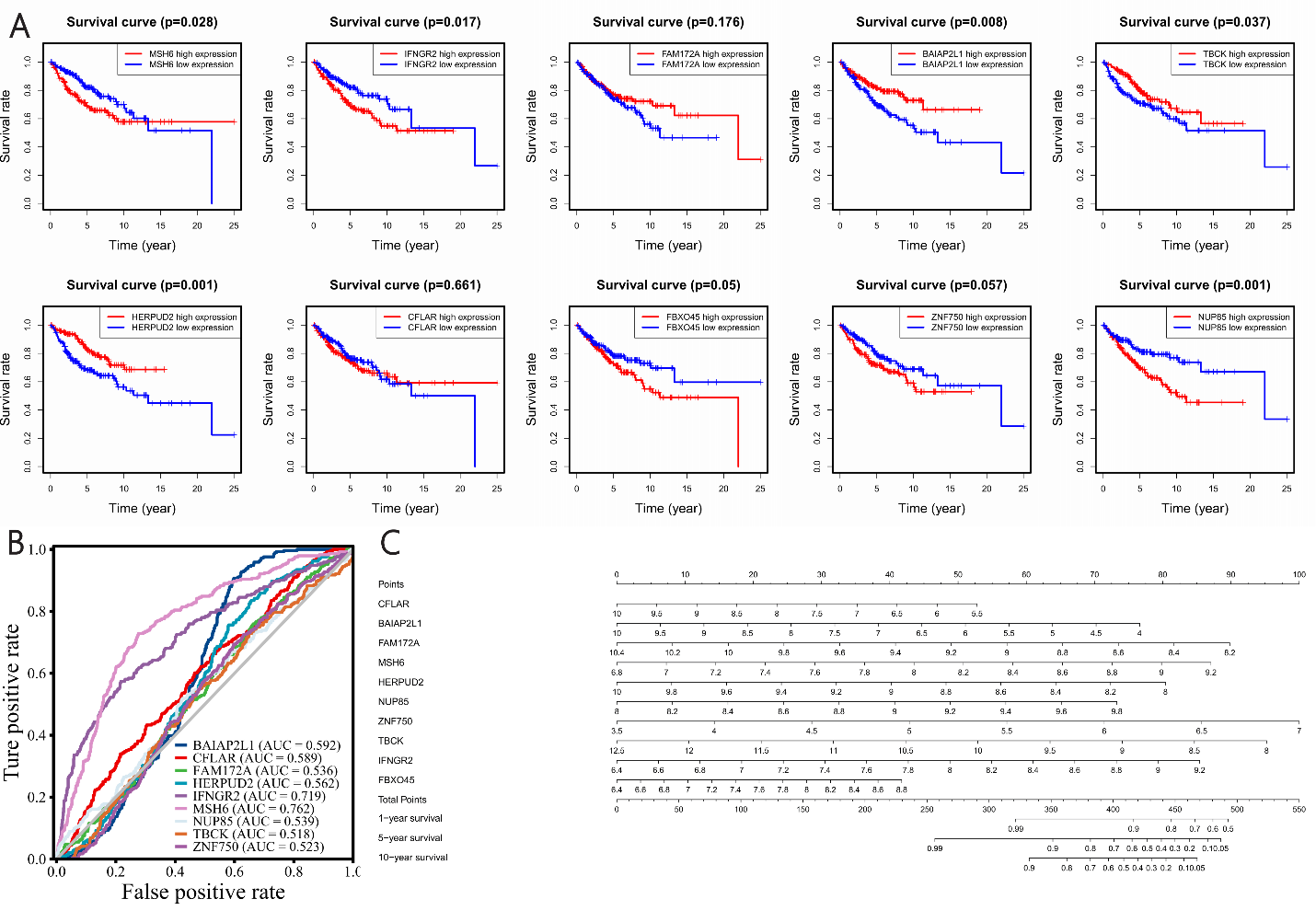
**

**Figure S5**. (A) Kaplan-Meier (KM) survival curves for hub-genes. (B) Receiver operational characteristic (ROC) curves were used to estimate the sensitivity and specificity of diagnostic in hub genes. (C) Nomograms constructed to predict 1-, 5-, and 10-year overall survival (OS) of Medulloblastoma (MB) patients in the training
